# Initial Experience of Intentional Internal High-Dose Policy Volumetric Modulated Arc Therapy of Neck Lymph Node Metastases ≥ 2 cm in Patients With Head and Neck Squamous Cell Carcinoma

**DOI:** 10.3389/fonc.2021.651409

**Published:** 2021-04-27

**Authors:** Tairo Kashihara, Satoshi Nakamura, Naoya Murakami, Kimiteru Ito, Yoshifumi Matsumoto, Kenya Kobayashi, Go Omura, Taisuke Mori, Yoshitaka Honma, Yuko Kubo, Hiroyuki Okamoto, Kana Takahashi, Koji Inaba, Kae Okuma, Hiroshi Igaki, Yuko Nakayama, Ken Kato, Fumihiko Matsumoto, Seiichi Yoshimoto, Jun Itami

**Affiliations:** ^1^ Department of Radiation Therapy in National Cancer Center Hospital, Tokyo, Japan; ^2^ Department of Radiology in National Cancer Center Hospital, Tokyo, Japan; ^3^ Department of Head and Neck Oncology in National Cancer Center Hospital, Tokyo, Japan; ^4^ Department of Pathology and Clinical Laboratories in National Cancer Center Hospital, Tokyo, Japan; ^5^ Department of Head and Neck Medical Oncology in National Cancer Center Hospital, Tokyo, Japan

**Keywords:** radiotherapy, volumetric modulated-arc therapy, simultaneous integrated boost, head and neck cancer, bulky tumors

## Abstract

**Background and Purpose:**

Most locoregional recurrences after definitive radiotherapy for head and neck squamous cell carcinoma (HNSCC) develop “in-field.” Dose escalation while sparing organs at risk can be a good solution for improving local control without increasing adverse effects. This study investigated the safety and effectiveness of volumetric modulated arc therapy (VMAT) using intentionally internal high-dose policy (IIHDP) to treat neck lymph node metastases (NLNM) ≥ 2 cm in HNSCC patients.

**Materials and Methods:**

We analyzed 71 NLNM from 51 HNSCC patients who had received definitive radiotherapy to treat NLNM ≥ 2 cm using the VMAT technique in our institution between February 2017 and August 2019. Thirty-seven NLNM from 25 patients were treated using IIHDP VMAT (group A), and 34 NLNM from 27 patients were treated with homogeneous-dose distribution policy (HDDP) VMAT (group B). One patient with three NLNM had one lymph node assigned to group A and the other two to group B. Adverse events and local recurrence-free survival (LRFS) was compared between the two groups.

**Results:**

In the median follow-up period of 527 days, there were no significant differences between the groups in terms of dermatitis or mucositis ≥ grade 2/3, but LRFS was significantly longer in group A (p = 0.007). In the Cox regression analysis after adjustment for the propensity score, group A also showed an apparently superior LFRS.

**Conclusion:**

Our initial experience of IIHDP VMAT suggested that IIHDP VMAT to treat HNSCC neck lymph node metastases measuring ≥ 2 cm was feasible and possibly led to better local control than HDDP VMAT.

## Introduction

Head and neck cancer is common worldwide, with more than 800,000 new cases and 400,000 deaths annually (1). Intensity-modulated radiotherapy (IMRT) and volumetric modulated arc therapy (VMAT) are widely used to treat head and neck cancer because they are associated with fewer adverse effects than conventional 3-dimensional (3D) radiation therapy (2–6). On the other hand, in GORTEC 2004-01 randomized phase III trial of locally advanced head and neck squamous cell carcinoma (HNSCC) patients, locoregional control was not improved with dose-escalated IMRT compared with 70 Gy in 35 fractions (7).

In contrast, a previous study reported a patient with bulky uterine cervical cancer who showed complete response using brachytherapy with increasing radiation dose inside the tumor, while sparing organs at risk (OARs) (8). This technique was named “intentional internal high dose policy (IIHDP)” brachytherapy and could be a new strategy for enhancing local tumor control without increasing treatment-related adverse effects.

In our institution, IIHDP VMAT has been applied to increase the dose delivered to bulky neck lymph node metastases ≥ 2 cm in head and neck squamous cell carcinoma (HNSCC). In the present study, to investigate the safety and effectiveness of IIHDP VMAT to treat neck lymph node metastases of HNSCC, we retrospectively compared neck lymph node metastases ≥ 2 cm treated with and without IIHDP VMAT.

## Materials and Methods

### Patient Population

The flowchart of patient inclusion is shown in **Table 1**. We conducted a retrospective review, from our institutional database, of the data of 394 patients with HNSCC who underwent radiotherapy using the VMAT technique to treat neck lymph node metastases in our institution between February 2017 and August 2019. Lymph node metastases were excluded if they measured < 2 cm on axial computed tomography (CT)/magnetic resonance imaging (MRI) images at the initiation of radiotherapy, or if they were irradiated with a prescription dose of more than 2 Gy in a fraction. Ultimately, 71 lymph node metastases from 51 patients were analyzed in detail. The diagnosis of lymph node metastasis was confirmed by biopsy or based on markedly increased uptake of ^18^F-fluorodeoxyglucose (FDG) in the area of lymph node metastases on positron emission tomography (PET). In total, 37 lymph node metastases from 25 patients were treated using IIHDP VMAT (group A), while 34 lymph node metastases from 27 patients were treated with homogeneous-dose distribution policy (HDDP) VMAT (group B). One patient with three lymph node metastases had one lymph node assigned to group A and the others to group B. The following factors were investigated in each group: age, sex, Zubrod performance status, body mass index, hemoglobin at the initiation of radiotherapy, albumin at the initiation of radiotherapy, primary site, the major axis of targeted lymph node metastasis (GTVn), TNM stage (based on the 8^th^ edition of the Union for International Cancer Control TNM staging system), TP53 mutation, postoperative recurrence, Brinkman index, drinking status, virus-associated tumor, concurrent drug administration, prescription radiation dose, radiotherapy durations, number of re-plannings and number of re-plannings due to tumor shrinkage. TNM stage was not investigated in patients with recurrent lymph node metastasis after surgery. Virus-associated tumors were defined as those associated with human papilloma virus (HPV) and Epstein-Barr virus (EBV). To detect HPV, p16 expression was evaluated using pathological specimens: strong, diffuse nuclear and cytoplasmic staining in 70% or more of the tumor cells was defined as p16 positivity (9). The patients’ EBV status was examined using paraffin section RNA *in situ* hybridization, which uses digoxigenin-labeled riboprobes to visualize EBV-encoded small RNA. Concurrent drug administration status was divided into three groups: chemoradiotherapy (with cisplatin or carboplatin), bioradiotherapy (with cetuximab), and radiotherapy alone.

**Table 1:** Flowchart of patient inclusion.

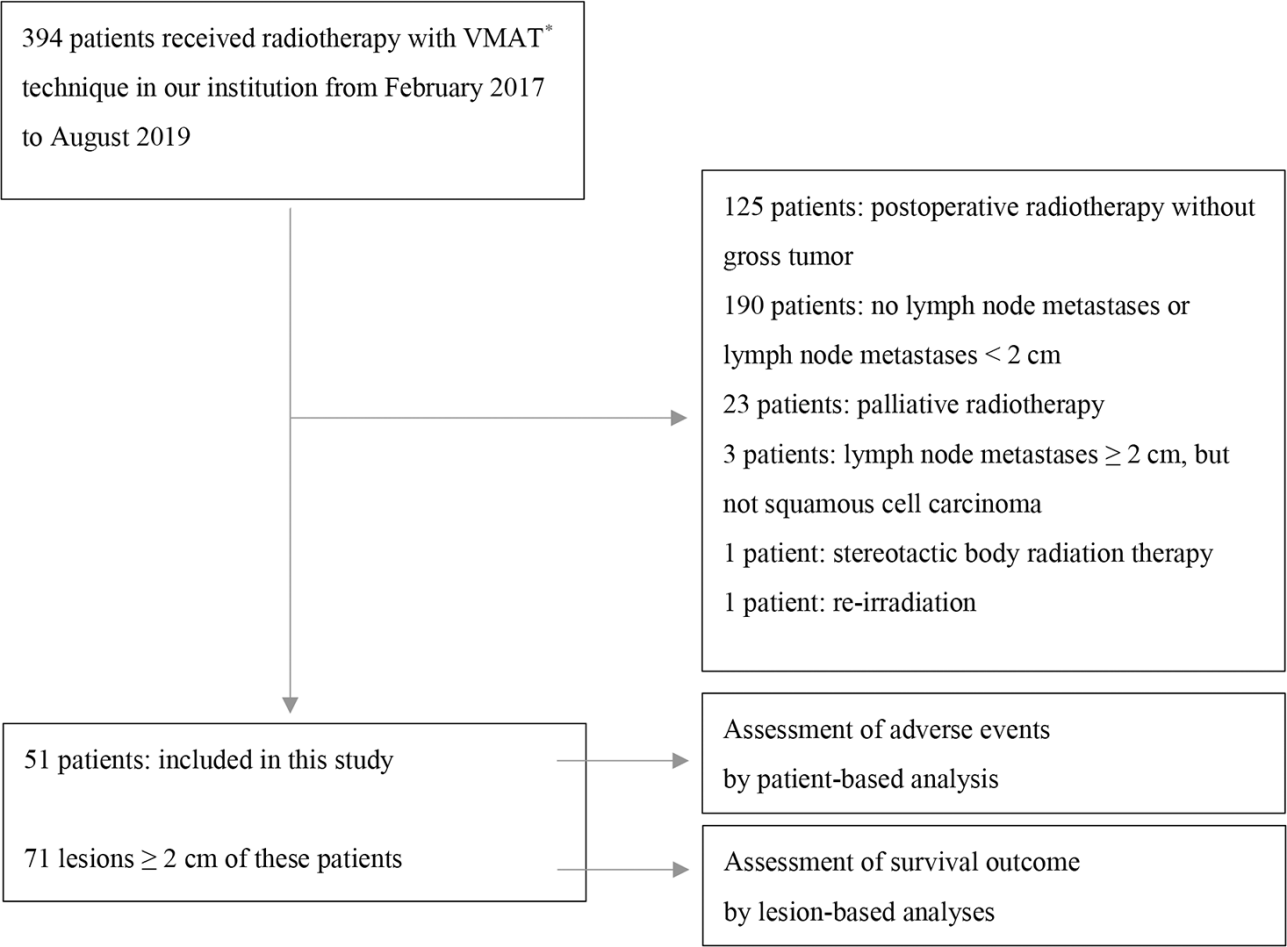	

VMAT^*^, volumetric modulated-arc therapy.

### Treatment Methods

The slice thickness of the CT images for treatment planning was 2 mm. Non-contrast CTs were used for treatment planning, and delineations were performed with reference to contrast CTs when the patients had no renal dysfunction or allergies to contrast agents.

Gross tumor volume of GTVn was contoured with reference to PET imaging and/or MRI. No clinical target volume margins were set, while the planning target volume (PTV) margins were set to 5 mm in all directions. When the PTV of the targeted lymph node metastasis (PTVn) extended outside the body, it was cropped to within 0–2 mm from the body surface. In group A, the IIHD area was contoured inside the lymph node and delivered 110% to 150% of the prescription dose as a guide that 1-2 mm needs for decreasing 10% dose. For example, a 110% IIHD area was contoured equal to or longer than 1–2 mm inside of GTVn. No PTV margins were set from the IIHD areas. All patients were treated using the VMAT technique at 2 Gy per fraction, once a day and five times per week. Radiotherapy was delivered using 6 MV X-rays from linear accelerators (Varian, Palo Alto, California, USA). Cone-beam CT images were taken more than once a week to allow CT-based guidance. When substantial shrinkage of the tumor or body shape occurred, re-planning was performed immediately to make an adaptive treatment plan. When the lymph node metastases had shrunk to less than 2 cm in diameter under IIHD treatment, the IIHD areas were erased. The IIHDP was applied when the attending physician judged that the lymph node metastases would not achieve complete response with HDDP VMAT, basing this judgment on the primary site and size of GTVn. The prescription dose basically covered 95% of the PTV. If necessary, the dose coverage on the PTV was reduced to spare OARs, while maximizing coverage as much as possible. Additionally, depending on the distance between the IIHD areas and the mucosal/skin surface, the planning risk volume (PRV) margin of these organs was set to more than 1 mm in all directions. The frequency of cone-beam image guidance was increased to daily or once every 2 days when the PRV margin was not sufficient. When cone-beam CT was taken, image-guided radiotherapy (IGRT) was subsequently administered based on the IIHD areas, PTV, and OARs. When no cone-beam CT images were taken, radiotherapy was performed based on the bony structures.

### Assessment of Outcomes

The effectiveness was investigated using lesion-based analysis, which accurately evaluates local effectiveness of IIHDP VMAT. Adverse events were evaluated using patient-based analysis because the locations of dermatitis and mucositis were not clear. Dermatitis and mucositis were evaluated by inspection and an endoscopy examination twice a week by two physicians during radiotherapy, and after the termination of radiotherapy, checked at least once a month for one year and after that, at least every 3 months. Acute dermatitis and mucositis were defined as those occurring within 3 months after the completion of radiotherapy. Acute dermatitis and mucositis, as well as late skin disorder and mucosal damage ≥ grade 2, were evaluated using the Common Terminology Criteria for Adverse Events ver. 5.0 in the both groups. Furthermore, we evaluated whether the observed adverse grade 3 effects were associated with IIHDP VMAT, in accordance with NCI guidelines for investigators. Specifically, in group A, we adhered to the adverse events reporting requirements of the NCI Division of Cancer Treatment, including those of the Diagnosis Cancer Therapy Evaluation Program and Cancer Imaging Program, as well as those of the Division of Cancer Prevention, such as Investigational New Drugs and Investigational Device Exemptions (definite, probable, possible, unlikely, unrelated). The one patient with lesions in both groups A and B was allocated to group A for adverse events assessment. Local recurrence-free survival (LRFS), disease-free survival (DFS), and OS were analyzed for all lesions. Local recurrences of irradiated lymph node metastases were defined as regrowth of the tumor, as was the detection of tumor cells upon biopsy or salvage operation, or markedly increased uptake of ^18^F-FDG in the areas of lymph node metastasis on PET. In case residual tumor was suspected, ^18^F-FDG-PET, CT, or MRI was taken to identify the viability of the tumor. When no tumor cells were detected on salvage operation specimens, the lymph node metastases were considered censored. However, when no residual tumor was detected in lesions that underwent biopsy, follow-up was continued. Addition to LPFS, true LPFS considering salvage surgery (tLPFS) was investigated. In this analysis, when there is no local recurrence after salvage surgery, the lesion is regarded as no local recurrence even if recurrence was detected in the surgical findings.

### Statistical Analyses

The distributions of patient and treatment characteristics were compared between the two groups. To compare categorical variables, the χ^2^ test was used, and continuous variables were compared using the Mann–Whitney U test. The frequencies of dermatitis and mucositis were compared between the two groups using logistic regression analysis. LRFS, DFS, and OS were estimated using the Kaplan–Meier method and compared using the log-rank test. Additionally, Cox regression analysis of LRFS was performed by applying propensity scores to adjust for differences in characteristics in 2 ways: (1) regression adjustment (2) stratification on the propensity score quintile. P-values < 0.05 were considered statistically significant. Statistical analyses were performed using IBM SPSS version 26 software (IBM Corp., Armonk, NY, USA).

## Results

### Patient/Treatment Characteristics

**Table 2** shows the patient and treatment characteristics by lesion-based grouping. There were 24 nasopharyngeal and 23 oropharyngeal cancer lesions. Fifteen lesions of 10 patients were recurrent lymph node metastases after prior operation. The total number of re-plannings was not significantly different between the two groups, but re-planning due to tumor shrinkage more frequently occurred in group A (p = 0.006). The dose-volume histogram analyses are shown in **Table 3**. The median volumes of the GTVn in groups A and B were 13.4 cm3 (range: 3.0–423.6 cm3) and 7.5 cm3 (range: 3.1–105.1 cm3) (p = 0.103), respectively. The maximal dose of GTVn was significantly higher in group A (*p* < 0.001). GTVn D98% was significantly higher in group A (p = 0.018), but PTVn D98% was not significantly different between the two groups (p = 0.067).

**Table 2:** Patient and treatment characteristics in group A and B with lesion-based grouping (71 neck lymph nodes).

Parameters	Group A (37 lesions)	Group B (34 lesions)	*p* value
	n (%)	n (%)	
Age, median (range)	63 (8-83)	63 (32-84)	0.549
Sex			0.823
Male	27/37 (73.0%)	24/34 (70.6%)	
Female	10/37 (27.0%)	10/34 (29.4%)	
Zubrod Performance status			0.376
0	26/37 (70.3%)	27/34 (79.4%)	
1	11/37 (29.7%)	7/34 (20.6%)	
Body mass index	20.5 (11.8-23.9)	19.8 (14.3-28.5)	0.454
Hemoglobin at the initiation of radiotherapy median (range)	13.7 (8.8-16.4)	13.8 (11.2-17.8)	0.991
Albumin at the initiation of radiotherapy median (range)	4.0 (3.3-4.8)	4.2 (3.3-4.8)	0.175
Primary site			0.429
Nasopharynx	9/37 (24.3%)	15/34 (44.1%)	0.078
Oropharynx	14/37 (37.8%)	9/34 (26.5%)	0.307
Hypopharynx	6/37 (16.2%)	5/34 (14.7%)	0.861
Larynx	2/37 (5.4%)	1/34 (2.9%)	0.606
Oral cavity	6/37 (16.2%)	3/34 (8.8%)	0.350
Maxillary sinus	0/37 (0.0%)	1/34 (2.9%)	0.293
The major axis diameter of GTVn median (range)	27 (20-82)	25 (20-70)	0.073
TNM stage (without recurrent patients, 27 and 29 lesions, respectively)			
T stage			0.225
1	6/27 (22.2%)	9/29 (31.0%)	
2	14/27 (51.9%)	8/29 (27.6%)	
3	3/27 (11.1%)	5/29 (17.2%)	
4	4/27 (14.8%)	7/29 (24.1%)	
N stage			0.588
1	8/27 (29.6%)	10/29 (34.5%)	
2	13/27 (48.1%)	11/29 (37.9%)	
3	6/27 (22.2%)	8/29 (27.6%)	
TP53 mutation, number			0.543
Wild type	21/37 (56.8%)	18/34 (52.9%)	
Mutation type	12/37 (32.4%)	14/34 (41.2%)	
Unknown	4/37 (10.8%)	2/34 (5.9%)	
Postoperative recurrence	9/37 (24.3%)	6/34 (17.6%)	0.491
BI^*^, median (range)	200 (0-1740)	80 (0-1200)	0.273
Drinking status, number			0.256
Yes	22/37 (59.5%)	23/34 (67.6%)	
No	14/37 (37.8%)	8/34 (23.5%)	
Unknown	1/37 (2.7%)	3/34 (8.8%)	
Virus associated	20/37 (54.1%)	22/34 (64.7%)	0.362
Concurrent drug administration			0.106
Chemoradiotherapy, number	35/37 (94.6%)	27/34 (79.4%)	
Bioradiotherapy, number	1/37 (2.7%)	6/34 (17.6%)	
Radiotherapy alone, number	1/37 (2.7%)	1/34 (2.9%)	
Prescription radiation dose			0.627
70 Gy in 35 fractions	35/37 (94.6%)	33/34 (97.1%)	
66 Gy in 33 fractions	1/37 (2.7%)	0/34 (0.0%)	
60 Gy in 30 fractions	1/37 (2.7%)	1/34 (2.9%)	
Radiotherapy durations (days), median (range)	52 (46-61)	51 (50-55)	0.064
With re-planning	29/37 (78.4%)	33/34 (97.1%)	0.445
With re-planning due to tumor shrinkage	15/37 (40.5%)	4/34 (11.8%)	**0.006**

BI, Brinkman index. Bolded text means that they have a statistically significant meaning.

**Table 3:** Dose-volume histogram analyses in group A and B.

Parameters, median (range)	Group A	Group B	*p* value
GTVn^*^ volume (cc)	13.0 (3.0-423.6)	4.5 (3.1-105.1)	0.103
IIHD^†^ area volume (cc)	1.7 (0.1-76.5)	0	N/A
GTVn max (%)	124.0 (112.2-173.9)	107.9 (104.9-111.4)	**<0.001**
GTVn D98% (%)	102.7 (100.7-108.0)	102.3 (98.0-104.2)	**0.018**
PTVn^‡^ D98% (%)	101.3(99.4-104.0)	102.0 (93.4-103.6)	0.067
PTVn D50% (%)	105.6 (104.2-115.2)	104.7 (100.3-107.2)	**<0.001**
PTVn RTOG^§^ Homogeneity index	1.240 (1.122-1.739)	1.093 (1.065-1.125)	**<0.001**
PTVn ICRU^¦^ Homogeneity index	0.179 (0.0733-0.6243)	0.0468 (0.0327-0.122)	**<0.001**

^*^GTVn, gross tumor volume of lymph node metastasis; ^†^IIHD, intentional internal high dose; ^‡^PTVn, planning target volume of lymph node metastasis; ^§^RTOG, Radiation Oncology Therapy Group; ^¦^ICRU, International Commission on Radiation Units and Measurements. Bolded text means that they have a statistically significant meaning.

### IIHDP VMAT Treatment in Group A

**Figure 1:**
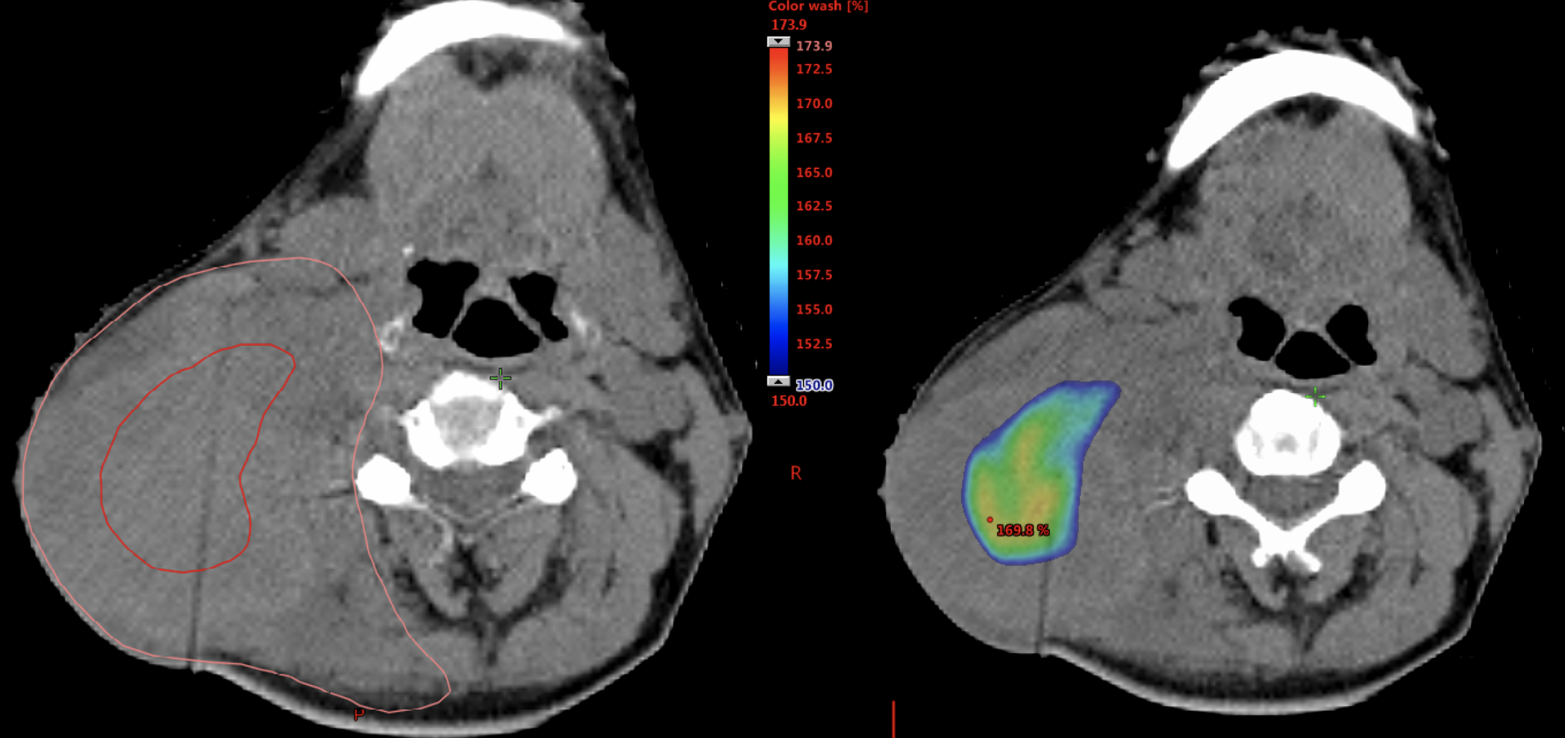
Example of the dose distribution of intentionally internal high dose policy (IIHDP) volumetric modulated arc therapy (VMAT) to treat nasopharyngeal cancer with massive neck lymph node metastases. In the left picture, delineations with red and orange lines represent intentionally internal high dose (IIHD) area and the clinical target volume (CTV), respectively. In the right picture, dose ≥ 150% of the prescription dose are shown as dose color wash.

The median IIHD volume was 1.7 cm3 (range: 0.1–76.5 cm3), and the median percentages of the volumes actually irradiated 110% dose to GTVn volumes were 41.2% (range: 12.5-92.0%). An example of IIHDP VMAT is shown in **Figure 1**. IIHDP VMAT was terminated after a median of 19 fractions (range: 7–33) in 18 patients. The median distances between the margins of the IIHD areas and the mucosal/skin surfaces were 1.95 cm (range: 0.31–4.92 cm) and 1.28 cm (range: 0.43–4.76 cm), respectively. Scheduled radiotherapy was completed in all 28 patients. Grade 3 mucositis unrelated to IIHDP radiotherapy was detected in six patients, while unrelated grade 3 dermatitis was found in one patient, and possibly related dermatitis in two patients.

### Assessment of Vessel Complications, Complications With Salvage Surgery, Skin Disorder and Mucosal Damage, and Survival Analysis

The median follow-up period was 729 days (range: 115–1459 days). The comparison of acute dermatitis and mucositis ≥ grade 2 is shown in **Table 4**. No significant difference was detected between the two groups. No late skin disorders or mucosal damage ≥ grade 2, or vessel complications were observed in any of the 51 patients. Furthermore, all the two patients who received salvage operations in group A experienced no complications. Nonetheless, two of five patients who received salvage operations in group B experienced complications with salvage operations; one patient developed postoperative wound infection, abscess formation, and tissue necrosis, and underwent reconstruction with a pectoralis major musculocutaneous flap, and the other patient suffered tracheal necrosis, fistula formation at the flap anastomosis, and underwent fistula closure with a pectoralis major musculocutaneous flap.

**Table 4:** Comparison of acute dermatitis and mucositis in group A and B (Patient-based analyses).

Parameters	n (%)	Odds ratio	95% confidence interval	*p* value
Dermatitis ≥ grade 2				
Group A	15/25 (60.0%)	0.553	0.170-1.798	0.324
Group B	19/26 (73.1%)	1.000 (reference)		
Dermatitis ≥ grade 3				
Group A	3/25 (12.0%)	0.573	0.121-2.702	0.481
Group B	5/26 (19.2%)	1.000 (reference)		
Mucositis ≥ grade 2				
Group A	15/25 (60.0%)	0.450	0.134-1.514	0.197
Group B	20/26 (76.9%)	1.000 (reference)		
Mucositis ≥ grade 3				
Group A	6/25 (24.0%)	1.053	0.289-3.840	0.938
Group B	6/26 (23.1%)	1.000 (reference)		

**Figure 2:**
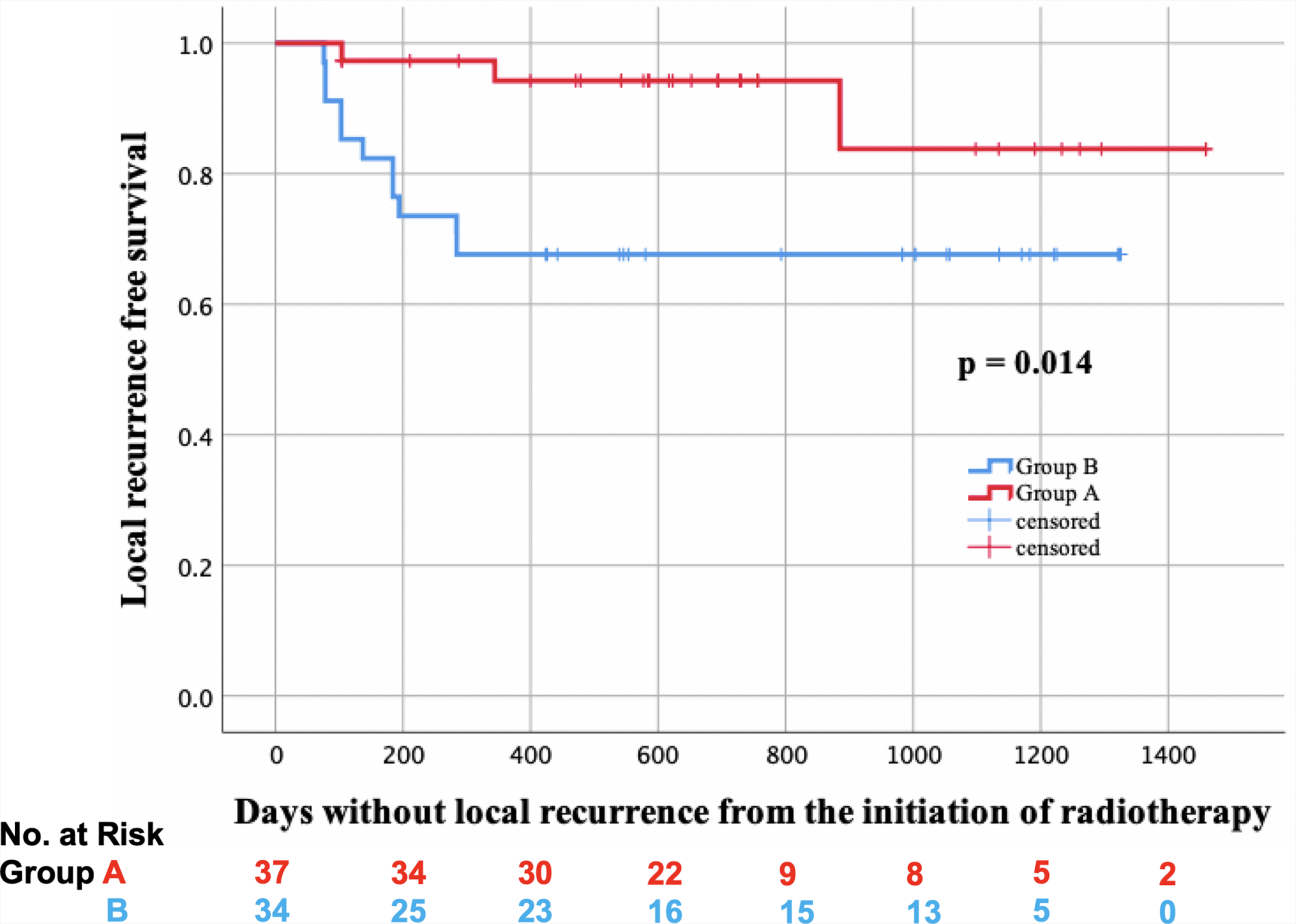
Local recurrence free survival (LRFS) in groups A (red line) and B (blue line).

**Figure 3:**
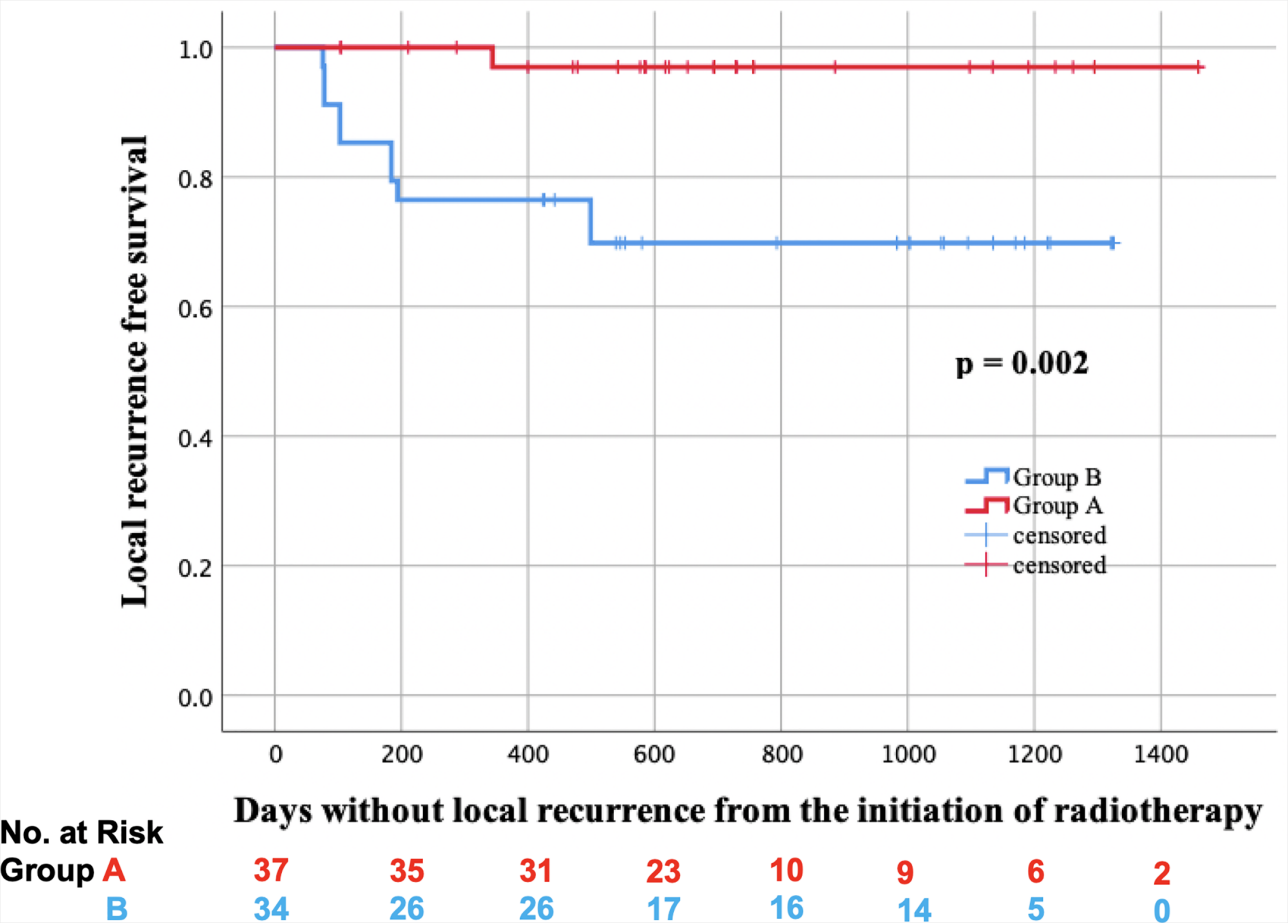
Local recurrence free survival considering salvage surgery (tLRFS) in groups A (red line) and B (blue line).

In total, two lesions received salvage surgery in group A, and residual tumors were detected in both of them. On the other hand, five lesions received salvage surgery in group B, and residual tumors were detected only in the two lesions of them. Additionally, biopsy was performed on the other six lesions in group A, but no residual tumors were found. The LRFS curves of groups A and B are shown in **Figure 2**. In the log-rank test, the LRFS was significantly longer in group A (p = 0.007). Additionally, in the Cox regression analysis of LRFS, with regression adjustment for propensity score and propensity score quintile stratification, an apparent advantage was observed in group A (OR: 0.121, 95% confidence interval [CI]: 0.024–0.599, p = 0.010; OR: 0.125, 95% CI: 0.026–0.596, p = 0.009). The propensity for receiving IIHDP VMAT was estimated using a multivariable logistic regression model that included baseline GTV volumes and primary site. Furthermore, tLRFS was significantly longer in group A (p = 0.002, **Figure 3**). In contrast, neither DFS nor OS were significantly different between the two groups (p = 0.954, 0.939).

## Discussion

In the present study, we investigated the safety and effectiveness of IIHDP VMAT for neck lymph node metastases of HNSCC that measured ≥ 2 cm. In patients who received IIHDP VMAT, LRFS was significantly longer, with no increase in adverse effects. This result was consistent after regression adjustment for propensity score and propensity score quintile stratification.

IIHDP radiotherapy was previously described by Kashihara et al. (8), who reported the case of a bulky uterine cervical neoplasm that was irradiated using IIHDP brachytherapy. The transverse diameter at the initiation of brachytherapy was 8.9 cm, but complete response was eventually obtained after treatment. Additionally, in treating bulky hepatocellular carcinoma, simultaneous integrated boost (SIB) with simultaneous integrated protection technique was reported by Crane et al. (10) This technique also applied dose escalation to tumors while protecting OARs. A retrospective analysis in patients treated using a similar technique for inoperable intrahepatic cholangiocarcinoma was reported by Tao et al. (11) In inoperable intrahepatic cholangiocarcinoma, improvements in local control and OS was observed when higher doses were delivered. On the other hand, in HNSCC patients, GORTEC 2004-01 randomized phase III trial reported that dose-escalated IMRT did not decrease locoregional recurrence in comparison with 70 Gy in 35 fractions (7). In dose-escalated IMRT group, a sequential boost of 25 Gy in 10 fractions to initial GTV was delivered after 50 Gy in 25 fractions to prophylactic PTV. Compared with this method, higher dose can be irradiated to the center of the tumors with IIHDP VMAT. Calculating the biologically effective dose to the center of the tumors with α/β = 10 Gy, the dose-escalated IMRT technique in GORTEC 2004-01 trial is 81 Gy, and IIHDP VMAT is 94 Gy (110%) to 137 Gy (150%). Therefore, IIHDP VMAT could be more effective technique for improving local control of the tumors.

Some previous studies have reported dose escalation using a VMAT technique in head and neck cancer (7, 12–17). Lauve et al. reported the results of the phase I trial of dose escalation to GTV in bulky HNSCC patients (12). Of 12 patients at dose level 2 (70.8 Gy in 30 fractions), 4 patients had Grade 3 acute skin toxicities, 6 had Grade 3 acute dysphagia, and 8 had Grade 3 acute mucositis. Furthermore, at dose level 3 (73.8 Gy in 30 fractions), radiotherapy was suspended due to premature Grade 3 dysphagia and mucositis in both of the 2 patients. Additionally, a Phase I/II study reported that 87% of the patients who received 67.2 Gy in 28 fractions for PTV had Grade 3 dysphagia (13). Dose escalation using HDDP VMAT could increase serious adverse effects because the definitions of irradiation increase the dose outside of the GTV and could increase the dose outside of the PTV. In the PET-guided focal-dose escalation study for HNSCC patients (14), although this technique was quite reasonable for improving tumor control, two patients experienced grade 4 adverse effects (one patient: dermatitis and the other: dysphagia) and one treatment related death was reported. In this study, PTV D98% was about 106% in dose level I and 111% of prescription dose in dose level II. On the other hand, in our study, PTVn D98% was not significantly increased (median, 101.3% in group A), indicating that the peripheral dose of PTVn area was not increased by using IIHDP VMAT (**Table 3**). This analysis means that OARs outside of the PTVn can be protected using IIHDP VMAT. However, GTVn D98% was significantly higher in group A, thus OARs inside of the PTVn may require more attention than was previously thought. No increase in adverse effects, such as dermatitis and mucositis, with the application of IIHDP was observed in the present study, but to increase the safety of this irradiation method, the GTVn D98% should be taken into consideration.

In many cases of bulky neck lymph node with a favorable response to radiotherapy, the inside of the tumor becomes necrotic and viable, contrast-enhanced lesions remain in the peripheral part of the bulky lymph nodes. In such circumstances, it is unknown why increasing the inner dose can contribute to controlling the bulky tumors, not by increasing the peripheral dose, but it might be related to hypoxia. Large tumors are associated with hypoxia inside of the tumor, which can weaken the effect of radiotherapy (18). In a randomized phase II trial of dose escalation using dynamic ^18^F-fluoromisonidazole PET/CT for locally advanced HNSCC patients, locoregional control was worse in patients with hypoxic tumors (15). An animal model has indicated that high dose irradiation to the hypoxic area inside of the tumor led to better local control (19). Additionally, RA Popple et al. reported that a modest boost dose (120-150% dose) to temporary hypoxic area would increase tumor control probability (20). It follows that IIHDP VMAT may be a reasonable technique for enhancing local control. Moreover, it is one of the strengths of IIHDP VMAT that no special image inspections are required such as ^18^F-FDG-PET/CT or ^18^F-fluoromisonidazole PET/CT.

In the patient whose one lymph node metastasis with a major axis diameter of 30 mm (lesion A) was treated using IIHDP VMAT (group A) and two lymph node metastases with major axes of 22 and 21 mm (lesion B_1_ and B_2_) were irradiated with HDDP VMAT (group B), complete response was seen in lesion A, while partial response was seen in lesions B_1_ and B_2_. Ten months after the completion of radiotherapy, ^18^F-FDG-PET MRI was taken to detect recurrent lesions. No increased uptake of ^18^F-FDG was seen in lesion A, but markedly increased uptake of ^18^F-FDG was seen in lesions B_1_ and B_2_, indicating that the IIHDP had been effective. Additionally, while the DFS was not related to IIHDP, the LRFS was significantly longer in group A, suggesting that IIHDP had local effectiveness.

The patient and treatment characteristics between the two groups were well-balanced, but there were marginally more patients with nasopharyngeal cancer in group B. Metastases from nasopharyngeal carcinoma are more likely to be controlled than those of other HNSCCs (21, 22). Despite this, local control of lymph node metastases was better in group A. Furthermore, nodal size and primary site are significant predictive factors of definitive radiotherapy in oropharyngeal and pharyngolaryngeal cancers (23, 24). Hence, these two factors were used to adjust patient characteristics with propensity score, and the results were consistent before and after the adjustment.

Regarding the number of re-plannings, no significant difference was found between the two groups. However, re-plannings due to tumor shrinkage were performed significantly more times in group A. This result implies that IIHDP VMAT conferred better tumor shrinkage during radiotherapy, leading to better LRFS in group A. To confirm this finding, additional research is needed.

In previous reports of head and neck cancer, IMRT using a SIB technique did not improve local control compared to 3D conformal radiotherapy and IMRT without a SIB technique, even though the PTV coverage was superior (25, 26). Likewise, in nasopharyngeal carcinoma, IMRT improved quality of life, but did not improve survival outcomes compared to 3D conformal radiotherapy (27). To the best of our knowledge, this was the first report on the effectiveness of dose escalation using a SIB-VMAT technique to treat neck lymph node metastases of HNSCC.

There were two limitations in the present study. Firstly, we could not evaluate whether mucosal dose and adverse effects increased or not by using IIHDP VMAT because a high dose was irradiated onto the primary tumor close to the metastatic lymph node. Nonetheless, the total number of mucositis cases with ≥ grade 2 or 3 was not larger in group A than in group B. In the present study, the IIHD area was contoured longer than 3 mm from the mucosal surface, so it is unlikely that any high dose was irradiated onto the mucosa. It can be used as a guide of a safe distance from the IIHD area to OARs. Secondly, this report was retrospective, with a small number of patients and a short follow-up period, thus it may have included some unknown biases. Especially, group A consists of patients who were selected at the discretion of the attending physician based on the lymph node size and primary site, thus this method introduces major selection bias. Additionally, most of the patients in group A received concurrent chemoradiotherapy, but in group B, only 79% of the patients received concurrent chemoradiotherapy, although the difference was not statistically significant. This difference could contribute to the better local control rate in group A. Moreover, because of a lack of standard dose escalation method, this study could be difficult to reproduce in other studies. Further additional research, including prospective trials that clearly defines the method of IIHDP to be reproducible, should solve this problem. The method would be to set the contouring of 110% IIHDP area 3-5mm inside the GTV, the contouring of 120% IIHDP area 3-5mm inside the 110% IIHDP area, increasing the internal dose by 10% every 3-5mm up to 150%. Furthermore, the locoregional recurrence survival should be assessed using tLPFS, which does not count as a recurrence if it can be salvaged by surgery, and it should be verified whether a dose escalation with IIHDP VMAT in a larger number of patients could outweigh the toxicity of selective neck dissection in patients without complete response with HDDP VMAT.

## Conclusion

Our initial experience of IIHDP VMAT suggested that IIHDP VMAT to treat HNSCC neck lymph node metastases measuring ≥ 2 cm was feasible and possibly led to better local control than HDDP VMAT. To verify the effectiveness of IIHDP VMAT, a large-scale prospective study that clearly defines the method of IIHDP to be reproducible would be required.

## Data Availability Statement

The raw data supporting the conclusions of this article will be made available by the authors, without undue reservation.

## Ethics Statement

The studies involving human participants were reviewed and approved by The Institutional Review Board of the National Cancer Center Hospital (approval number, 2017-091). The patients/participants provided their written informed consent to participate in this study. Written informed consent was obtained from the individual(s) for the publication of any potentially identifiable images or data included in this article.

## Author Contributions

Conception and design of the work: TK, SN, NM, KiI, YM, KKa, GO, TM, YH, YK, HO, KT, KoI, KO, HI, YN, KKo, FM, SY, JI. Supervision and wrote the paper: TK. All authors contributed to the article and approved the submitted version.

## Conflict of Interest

YN reports personal fees from AstraZeneca, outside the submitted work. YK reports grants from Canon Medical Systems, outside the submitted work. KI reports grants from Elekta K.K., outside the submitted work. HI reports personal fees from Itochu, personal fees from ViewRay Inc., grants from HekaBio, outside the submitted work. JI reports grants and non-financial support from KeyJ, personal fees from Alpha Tau, personal fees from ItoChu, outside the submitted work.

The remaining authors declare that the research was conducted in the absence of any commercial or financial relationships that could be construed as a potential conflict of interest.

## Abbreviations

CT, computed tomography; CTCAE the Common Terminology Criteria for Adverse Events; CTV, clinical target volume; EBER, Epstein-Barr virus-encoded small ribonucleic acid; EBV, Epstein-Barr virus; FDG, fluorodeoxyglucose; GTV, gross tumor volume; HDDP, homogeneous-dose distribution policy; HNSCC, head and neck squamous cell carcinoma; HPV, human papilloma virus; IIHD, intentional internal high dose; IIHDP, intentionally internal high dose policy; IMRT, intensity-modulated Radiotherapy; ISH, *in situ* hybridization; LRFS, local recurrence free survival; MRI, magnetic resonance imaging; OAR, organ at risk; PET, positron emission tomography; PRV, planning risk volume; PTV, planning target volume; RNA, ribonucleic acid; SIB, simultaneous integrated boost; VMAT, volumetric modulated-arc therapy.
